# Effect of Ultrasonic Vibration on Interlayer Adhesion in Fused Filament Fabrication 3D Printed ABS

**DOI:** 10.3390/polym11020315

**Published:** 2019-02-13

**Authors:** Alireza Tofangchi, Pu Han, Julio Izquierdo, Adithya Iyengar, Keng Hsu

**Affiliations:** Department of Mechanical Engineering, University of Louisville, Louisville, KY 40292, USA; alireza.tofangchi@louisville.edu (A.T.); pu.han@louisville.edu (P.H.); julio.izquierdo@louisville.edu (J.I.); adithya.iyengar@louisville.edu (A.I.)

**Keywords:** fused deposition modeling, Fused Filament Fabrication, ultrasonic vibrations, inter-layer strength, inter-layer adhesion

## Abstract

One of the fundamental issues in the Fused Filament Fabrication (FFF) additive manufacturing process lies in the mechanical property anisotropy where the strength of the FFF-3D printed part in the build-direction can be significantly lower than that in other directions. The physical phenomenon that governs this issue is the coupled effect of macroscopic thermal mechanical issues associated with the thermal history of the interface, and the microscopic effect of the polymer microstructure and mass transfer across interfaces. In this study it was found that the use of 34.4 kHz ultrasonic vibrations during FFF-3D printing results in an increase of up to 10% in the interlayer adhesion in Acrylonitrile Butadiene Styrene (ABS), comparing the printing in identical thermal conditions to that in conventional FFF printing. This increase in the interlayer adhesion strength is attributed to the increase in polymer reptation due to ultrasonic vibration-induced relaxation of the polymer chains from secondary interactions in the interface regions.

## 1. Introduction

Fused Filament Fabrication (FFF) technology represents a capable, flexible, and cost-effective option in the additive manufacturing industry. Although currently a widely adopted prototyping tool in production of engineering products, for the FFF process to advance into a manufacturing tool, its process and material characteristics, such as tolerance and accuracy, surface finish, as well as material property uniformity, need to reach a high level of maturity. Specifically, work has been reported on optimization of FFF process parameters such as temperature, print path strategy, layer height, and over extrusion to remove defects between printed tracks [1,2,3,4,5] with statistical tools incorporated [6,7,8,9,10,11]. Optimization-based approaches, though, address certain issues with the FFF process, they compromise on others when the physical mechanisms that govern those properties require changes of a given parameter in different ways [12]. One example is that printing at higher nozzle temperatures results in increasing part strength isotropy, but it reduces dimensional and geometrical tolerances. 

Specific to material property uniformity, the tensile strength of as-built FFF parts in the inter-layer direction (typically referred to as the build-direction) typically falls in the range of 10–65% of that in the direction along the filaments depending on process conditions and materials [13,14]. Unlike the strength in directions normal to the build direction which can be optimized by infill and raster strategy, the inter-layer strength of the FFF part is governed by the thermal history-dependent mass transfer across layers as well as the rheology-dependent microstructures of the printed tracks [13,15,16,17]. The polymer chains on two sides of an interface go through three stages of wetting, diffusion, and randomization before the interface between the two sides of the polymer heals and the mechanical property in the interlayer direction reaches near to that in the direction of the filaments. At this point, part property becomes isotropic or uniform in any given direction. The rheology-dependent microstructure effects also play a key role in interface healing in that the microstructure of the polymer around the interface has a strong effect on the diffusivity of the polymer [18,19,20,21,22,23,24]. It is possible that although increasing the time during which the interface stays above the critical temperature can improve the healing process, increasing diffusivity will provide a similar effect of improving interface healing.

Several techniques have been demonstrated to effectively improve the interlayer bond strength in FFF printed materials by introducing additional heating to the interfaces either as a post-fabrication process, or an in-process technique. The objective is to increase mass transfer on the interface by increasing the temperature dependent diffusivity. Additives to the surface of filaments were also used which were later used as a local energy coupling sources for local heat generation to promote polymer diffusion [25,26,27]. Infrared heating and laser heating were used to introduce additional heat to the printed surface just prior to deposition of the current layer to increase the interface temperature and therefore interlayer adhesion [28,29,30].

From the same correlation, it is also evident that reducing the radius of gyration of polymer chains could also result in similar improvement in inter-layer strength. This can be achieved by promoting relaxation of the stretched polymer chains in the printed tracks. The work reported here describes, for the first time, a technique of using ultrasonic vibrations as an in-process method to reduce the chain–chain secondary interaction and allow more relaxation and diffusion of the polymer in the interface region to result in an improvement in interfacial adhesion strength. This effective technique has the potential to produce FFF-printed parts with isotropic mechanical properties.

## 2. Experimental

### 2.1. Sample Preparation

**Figure 1:**
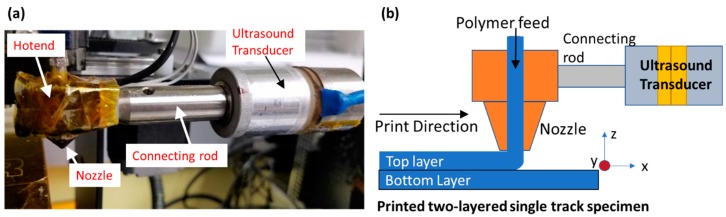
Laboratory-implemented apparatus for incorporating ultrasonic vibrations into Fused Filament Fabrication (FFF)-deposition of thermoplastic polymer. (**a**) photogragh of actual apparatus. (**b**) Conceptual sketch.

Shown in Figure 1 is the configuration where two-layered single-track specimens were printed on a gantry style 3D printer (MakerGear M2, MakerGear LLC, Beachwood, OH, USA), modified to allow ultrasound vibrations to be incorporated into the extruded filament. Each specimen has two single tracks one on top of the other. Acrylonitrile Butadiene Styrene (ABS) filament feedstocks of 1.75 mm-diameter and a 0.87 mm-diameter nozzle (measured using optical microscope, Hatchbox 3D, Pomona, CA, USA) were used for printing all specimens. The temperatures for the extruder/build plate and print speed settings were 190 °C/90 °C at 200 mm/min respectively. Both top and bottom tracks in a given specimen were printed in the same direction (from +*x* to −*x*, parallel to the longitudinal axes of the transducer) in the same length (30 mm). Track nominal widths (denoted as “b”) were varied from 0.63 to 1.27 mm with a fixed thickness (denoted as “h”) of 0.35 mm. To ensure the bottom (first-layer) track reaches a uniform spatial temperature distribution along its entire length prior to the printing of the top layer (second-layer) track, sufficient pause time (~31 s) was introduced between the printing of the two layers by adding a single layer, single line loop around the bottom track. Selection of the pause time was based on IR thermography where the first layer reaches a steady state at the build plate temperature. In ultrasound-assisted specimens, the ultrasound was only applied during the printing of the top track. The bottom tracks were identical in all specimens within each set.

### 2.2. Ultrasound-Assisted FFF Printhead

Shown in Figure 1 are a conceptual sketch and photograph of the actual apparatus implemented. A 40 kHz-piezoelectric crystal-based bolt-clamped transducer with power rated at 25 W was connected to the heater block at the hotend section of the printhead using a 304 stainless-steel (Thorlabs, Cleveland, OH, USA) connecting rod and threaded fasteners. The use of the connecting rod allows for the coupling of vibrations generated by the transducer, and the reduction of heat transfer from the heater block into the transducer. With the hotend section mounted on the printhead assembly, the overall resonant frequency of the printhead was measured to be 34.4 kHz. At this frequency, the maximum peak-to-peak amplitude was measured as 0.8 micrometers using a single point laser Doppler vibrometer (OMS, Laguna Hills, CA, USA) at 25 W-power input from the generator into the transducer (limited by the maximum amplitude of the transducer used). A frequency-adjustable ultrasonic signal generator was adjusted to the resonant frequency of the printhead and connected to the transducer to provide excitation signals that allowed the nozzle surface to vibrate at the measured frequency. This resonant frequency is determined by measuring the maximum vibration amplitude at the surface of the nozzle while sweeping the generator frequency. When excited at frequencies away from the measured resonance, the transducer output is instead dissipated in the entire transducer–connecting rod–hotend assembly as heat. In order to de-couple the effect of heat from ultrasonic vibrations on the rheology and interlayer-adhesion, out-of-resonance excitation conditions are avoided. In addition, to examine the effect of frequency, modifications to the physical system would be required. Therefore, the effect of frequency is not included in the scope of this work.

To prepare each specimen printed with ultrasound assistance, the transducer was turned on just before the top track deposition started. The print conditions and parameters in all samples with ultrasound assistance were identical to those in the control specimens where no ultrasound was applied. 

### 2.3. Interfacial Adhesion Characterization—Trouser Peel Test

The separation/peeling forces and the corresponding adhesion energy of the bonds between the printed two-layer tracks were measured by adopting the technique of the ASTM F88 Peel Test. This standard system is typically used to measure the adhesive force between two sealed flexible bands. This is an appropriate method of testing adhesion energy in this study since it applies to flexible specimens, allowing for a spatial view of adhesion between two layers (as compared with tensile testing where the measured strength corresponds to the lowest of a series of interfaces).

**Figure 2:**
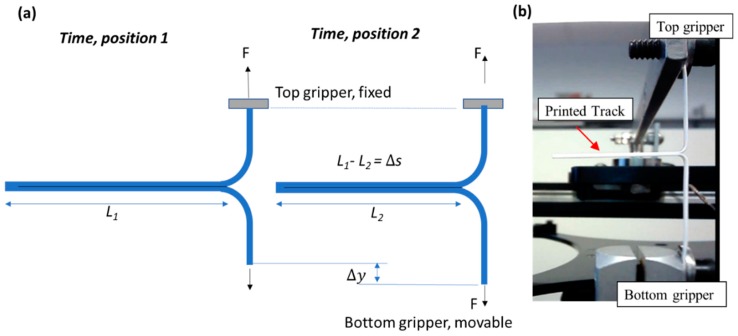
Trouser peel test of two-layer single track specimens for interfacial adhesion strength testing. (**a**) concept sketch, (**b**) actual peel test of printed double track specimens.

Figure 2 shows how this method was implemented. An initial cleft is introduced between the two adhesive layers which are pulled in opposite directions at constant rate while the peel force is continuously recorded as the cleft progresses towards the other end. Specific to the study reported here, a small pre-crack or slit between the top and bottom tracks was introduced by applying an adhesion barrier on the end of the first track. Black ink from a permanent marker was used as the adhesion barrier to prevent bond formation with the two layers, and therefore left a small “pre-crack” on the interface. During each trouser peel test, the two free ends of a specimen were mounted on the grippers of a lab-built trouser peel test apparatus. The gripper travel rate was set to 30 mm/min for all specimens while the curvature of the pulled tracks remained fairly unchanged during the peel test. Since only one gripper end moved, kinematically this resulted in a peeling rate equal to half of that of the gripper speed, i.e., 15 mm/min. 

On the lab-built trouser peel test apparatus a 200-Newton capacity load cell (Omega) was used to measure the peeling force. A mechanical lever was used to transfer the peeling force to the load cell. Outputs from the load cell were recorded in real-time during each peel test. Each data point in the plots was calculated by obtaining force-deflection (*F* vs. Δ*s*) values for four sample specimens with identical print setting, which then were averaged out to obtain the adhesion strength value assigned for that particular size.

### 2.4. Calculation of Adhesion Energy (R)

Based on the principle of conservation of energy in Figure 2, the balance between the external mechanical work, adhesion energy of the tracks, and the strain energy needed to bend the tracks can be expressed as:

External mechanical work = Adhesion Energy + Strain energy, or

(1) $$F\Delta_{y}=R\left(\Delta_{s}b\right)+\Delta U_{\varepsilon }$$

where *F* is the measured force and b is the width of the printed two-layer track specimens, $\Delta_{y}$ is the point load displacement, *R* is the adhesion energy [$\frac{J}{m^{2}}$ or $\frac{N}{m}$], $\Delta_{s}$ is the interface separation distance, and $\Delta U_{\varepsilon }$ is the strain energy. By manipulating the energy balance, one arrives at

(2) $$R=\frac{F}{b}\left(\frac{\Delta_{y}}{\Delta_{s}}\right)-\frac{\Delta U_{\varepsilon }}{b\Delta_{s}}$$

Based on the assumption for thin layers [31] where the bending effects are negligible, $(\Delta U_{\varepsilon }\approx 0$), and that the curvature of tracks remained unchanged and that track separation was created by downward motion of the single gripper, ($\frac{\Delta_{y}}{\Delta_{s}}\approx$ 2), the adhesion between the top and the bottom layer can be expressed as

(3) $$R=\frac{2F}{b}$$

By measuring *F* and *b*, the spatially resolved interfacial energy can be calculated and obtained. Practically, the average recorded value of *F* along the track is used to calculate the adhesion strength of the track. The authors also note that the *b* here which refers to the width of the printed tracks, can deviate from the actual width of the interface due to the printed track geometry. A calibration process (described below) is used to correct the geometrical deviation in each specimen. The calculated and presented *R* values hereafter have been corrected using this process. 

### 2.5. Geometrical Correction and Calibration

**Figure 3:**
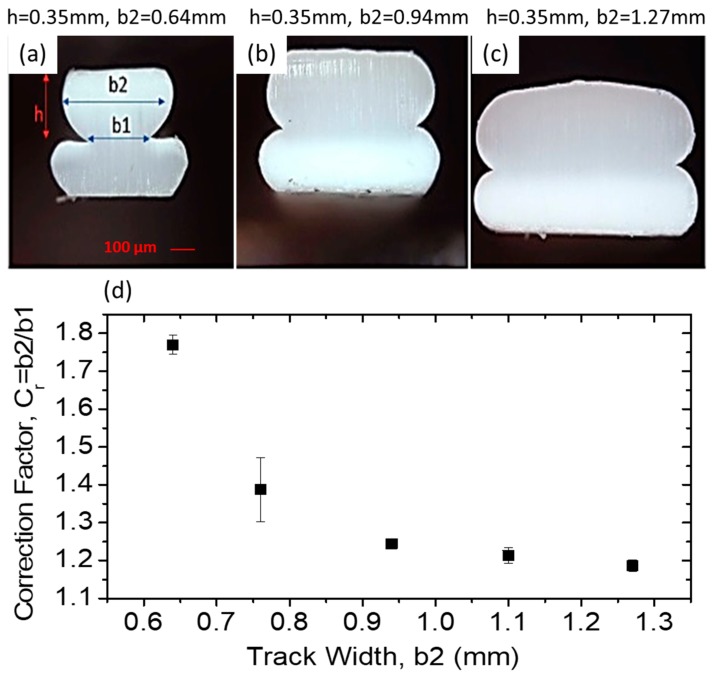
Effect of printed track geometry and corresponding correction factor for adhesion calculation. (**a**–**c**) double-layer track specimens at various widths, (**d**) correction factors applied for adhesion calculations for *h* = 0.35 mm with varying width. The errors bar represent the standard deviation measured at four sections for each given track width.

As shown in Figure 3, due to the cross-sectional geometry of printed tracks, the actual layer width at the interface (*b*1) is smaller than that of the nominal width of the layer (*b*2). To correct this to use in the adhesion equation, a series of systematic tests was conducted on several tracks with exact print conditions to estimate the actual values of *b*1 and *b*2. 

Figure 3a–c shows three representative images of track cross section for fixed height (0.35 mm) and varying width (*b*2 = 0.64 to 1.27 mm). Note that here the height only refers to the thickness of the 2nd layer track, as the 1st layer track thickness was set to be 15% less than that of the second layer to ensure the bottom of the 2nd layer track was fully within the top surface of the 1st layer track. 

It is evident in Figure 3 that the nominal width of each track (*b*2, manually measured by caliper) is greater than the width of the actual interface (*b*1, measured optically). We define correction factor $=\frac{b2}{b1}$, where its variation as a function of nominal width is plotted in Figure 3d. Each data point in the plots was averaged out based on measured values of *b*2 and *b*1 at four different cross-sections. 

The correction factor Cr was used directly to calculate values of adhesion ($R=\frac{2F}{b1}$) by starting with the nominal track size (*b*2) and multiplying that by the corresponding Cr for that size ($R=\;\frac{2F}{b2}\times Cr=\frac{2F}{b1})$, giving the true value of adhesion based on the actual interface width. 

## 3. Results and Discussion

**Figure 4:**
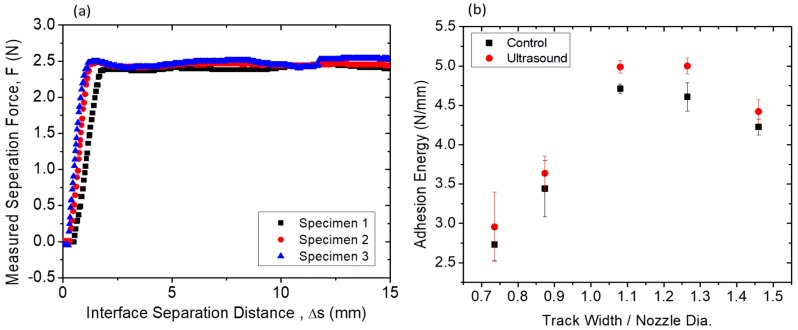
(**a**) Force-Separation distance curves for 1.0 mm-wide example peel tests (**b**) Interfacial adhesion strength dependence on width of printed tracks for control and ultrasonic vibration-assisted FFF conditions. The error bar represents the standard deviation of adhesion strength measured for four test specimens at a given track width, both in control and ultrasound condition.

It can be seen in Figure 4a that the adhesion forces along a given specimen remain consistent. It is also observable that across different specimens printed in identical conditions, the adhesion also remains consistent. In Figure 4b one can observe that when the track width-to-nozzle diameter ratio varies from 0.74 to 1.47 (track width 0.64 to 1.27 mm), the interfacial adhesion initially increases but plateaus as the ratio reaches around 1.2, and then decreases at larger ratios. The authors attribute this behavior to changes in the local shear forces experienced by the polymer in the flow as it transitions from flow through the cylindrical nozzle to flow in the horizontal direction along the tracks. As the track width increases, the overall flow rate, and therefore flow velocity, of the polymer increases. This increase in flow velocity in turn increases the local shear stresses experienced by the polymer near the top and bottom surfaces of the track and causes an increased amount of disentanglement of polymer chains in those regions. This increase in chain alignment (or decrease in entanglement of polymer chains) can increase the diffusivity of the polymer in the orthogonal directions [19], which is conducive to an increase in interlayer adhesion due to increased reptation of the polymer across the interface.

Although the increase in polymer chain disentanglement can result in an increase in diffusivity, it also increases the amount of chain relaxation from the stretched states needed to allow entanglement of newly diffused chains. This effect counteracts that of the increase in polymer reptation. It is reasonable to expect that the result is an increase in the interlayer adhesion as the width increases, but the adhesion would eventually level off at high degrees of polymer stretching. 

In the presence of this excessive shear flow stress and chain domain stretching, the viscoelastic behavior of the extruded polymer-melt and the rapid cooling in the newly formed interfaces after the polymer exits the nozzle cause residual tensile stresses to be built up in the vicinity of the interfaces. It has been shown that the cooling rates in FFF printing environments similar to those used in the current study are in the 10–20 degrees per second range [29], and a printed track can cool from above 150 °C to below 40 °C in approximately 10 s. In fact, during trouser peel tests, when the sum of these residual stresses (due to fast cooling) and the applied mechanical stresses (due to mechanical pulling) on the interface reach the strength of the interface, the interface tears. Therefore, as the proportion of shear flow stress is higher in wider tracks (track width/nozzle dia > 1.2), the share of the required mechanical stress (from pulling) in order to exceed the strength on the interface reduces, as does the adhesion strength.

The addition of ultrasound at 25 W power produces observable effects on the interfacial adhesion. It can be seen from Figure 4 that across the tested range of track width–nozzle diameter ratios, the use of ultrasound causes up to 10% increase in the interfacial adhesion measured in the trouser peel tests. This increase is not due to fluctuations in the temperature as the both the built-in thermistor was used to monitor the hotend temperature and the fluctuation remained within 1 °C during printing. The authors attribute the observed increase to the kinetic effects of ultrasound on the polymer. The correlation between the interfacial adhesion strength and the mass transfer and microstructure of the polymer can be described by the relation proposed by Ezekoye et al. [32]:

(4) σtσmax=(tweldτrep)14=(tweldDsRg2)14

where $\sigma_{t}$, $\sigma_{max}$ are the strength of the interface and the tensile strength of the material respectively, $t_{weld}$ is the healing time of the interface (or time during which the interface stays above the glass transition or melting temperature), $\tau_{rep}$ is the reptation time, $D_{s}$ is the center of mass diffusivity of the polymer chains, and $R_{g}$ is the radius of gyration of the polymer chains. The radius of gyration describes how stretched the polymer chains become as the polymer goes through the two required steps of change in the flow: the reduction of the flow diameter from that of the feedstock to the size of the nozzle opening, and the overall 90-degree turn the polymer flow makes as it exits the nozzle and forms the printed track. The radius of gyration describes the microstructure of polymer near the interfaces which plays a key role in determining the local diffusivity as well.

In the kinetic effect, as ultrasound propagates in a polymer melt in the form of pressure waves, the dynamic pressure gradients in the polymer melt raise the energy state of the polymer chains. The effect is similar to that of increasing the temperature of the polymer chains: it allows for a larger extent of relaxation from secondary interactions between the chains. One of the resulting effects is a reduction in viscosity of the polymer melt [33,34,35,36,37,38]. In the study described here, the increased relaxation plays a key role in the reptation and changes of the polymer chain conformation from the stretched states due to the extrusion of polymer melt in FFF. As relaxation of the secondary interactions increases, reptation and therefore polymer diffusivity increases, which gives rise to higher interfacial adhesion. In addition, increased relaxation from chain–chain secondary interactions also allows for relaxation of the polymer chains from the stretched states to conformations that are less stretched, or a reduction in the radius of gyration, $R_{g}$. While both effects enable an increase in the interfacial healing process, the process of reducing the radius of gyration of the polymer chains is also associated with entanglement of the polymer chains across the interface. The authors attribute the increase in interfacial adhesion observed here when ultrasound assistance is used to this effect. Unlike temperature increase, which despite allowing for more diffusion and therefore increasing inter-layer bond strength but causing issues in dimension and geometry control, ultrasound-based techniques such as the one examined in this study can decouple the thermal mechanical effects from the rheological effect. In essence, it represents a feasible approach to improving the mechanical properties of FFF-printed materials.

Figure 4b also shows that as the track width–nozzle ratio increases, the effect of ultrasonic vibrations becomes more consistent and observable. The authors attribute this to the increases in the relaxation of the stretched polymer chains during higher shear as the width increases. As discussed earlier, ultrasonic vibration reduces polymer melt viscosity due to increased relaxation, it is conceivable that as the shear in the polymer increases due to the larger track widths at the same height, the increased polymer stretching would also allow an increase in relaxation. In the three-stages of the polymer interface healing process, increased relaxation combined with increased reptation results in an increasing degree of entanglement in the interface region. The result is an increase in the interfacial adhesion. As eluded in the experimental section, in order to de-couple the effect of heat from ultrasonic vibrations on the rheology and interlayer-adhesion, out-of-resonance excitation conditions are avoided. Therefore, to examine the effect of frequency, modifications to the physical system to ensure resonance is required. This is currently being investigated by the authors. As part of on-going work, both longitudinal and transverse modes of vibration will be investigated. The effect of frequency, power, and material on the microstructure of the polymer near the interfacial regions will also be investigated.

## 4. Conclusions

The effect of ultrasonic vibration on the interfacial adhesion strength of 3D printed material using Fused Filament Fabrication (FFF) was investigated. Two-layer single track trouser peel tests were performed on FFF-3D printed ABS specimens in control and in ultrasound vibration-assisted conditions. The effects of track width in the printed tracks on the shear and microstructure of the polymer and therefore reptation and interfacial adhesion were observed and discussed. An increase of up to 10% in the interlayer adhesion was found in FFF-printed specimens when ultrasonic vibrations were incorporated into the extrusion path of the FFF printing. The effects associated with the use of ultrasound vibrations are attributed to the ultrasound-induced increase in polymer chain relaxation from secondary interaction, and the resulting increase in reptation and entanglement from stretched states.
